# Prolyl 4-Hydroxylase Domain Protein 3-Inhibited Smooth-Muscle-Cell Dedifferentiation Improves Cardiac Perivascular Fibrosis Induced by Obstructive Sleep Apnea

**DOI:** 10.1155/2019/9174218

**Published:** 2019-06-27

**Authors:** Jiayi Tong, Fu-chao Yu, Yang Li, Qin Wei, Chen Li, Penghao Zhen, Guanghao Zhang

**Affiliations:** ^1^Southeast University, China; ^2^Department of Cardiology, Zhongda Hospital of Southeast University, China; ^3^Department of Cardiology, The Second Hospital of Shandong University, China

## Abstract

**Background:**

Intermittent hypoxia (IH) induced by obstructive sleep apnea (OSA) is a leading factor affecting cardiovascular fibrosis. Under IH condition, smooth muscle cells (SMAs) respond by dedifferentiation, which is associated with vascular remodelling. The expression of prolyl 4-hydroxylase domain protein 3 (PHD3) increases under hypoxia. However, the role of PHD3 in OSA-induced SMA dedifferentiation and cardiovascular fibrosis remains uncertain.

**Methods:**

We explored the mechanism of cardiovascular remodelling in C57BL/6 mice exposed to IH for 3 months and investigated the mechanism of PHD3 in improving the remodelling in vivo and vitro.

**Results:**

In vivo remodelling showed that IH induced cardiovascular fibrosis via SMC dedifferentiation and that fibrosis improved when PHD3 was overexpressed. In vitro remodelling showed that IH induced SMA dedifferentiation, which secretes much collagen I. PHD3 overexpression in cultured SMCs reversed the dedifferentiation by degrading and inactivating HIF-1*α*.

**Conclusion:**

OSA-induced cardiovascular fibrosis was associated with SMC dedifferentiation, and PHD3 overexpression may benefit its prevention by reversing the dedifferentiation. Therefore, PHD3 overexpression has therapeutic potential in disease treatment.

## 1. Background

Obstructive sleep apnea (OSA) is a common disorder which is characterized by total or partial collapse of upper airways alternating with normal breathing [1, 2]. Obstruction in gas exchange leads to chronic intermittent hypoxia (CIH), oxygen desaturation, hypercapnia, and arousal [3, 4]. The Wisconsin Sleep Cohort Study reported that OSA prevalence was 4% in middle-aged men and 2% in middle-aged women (age 30–60 years) [5]. Subsequent studies suggest that prevalence is higher than previously reported in high-income countries (10% in women and 20% in men) [6, 7]. In consideration of the obesity pandemic, the number of cases of OSA is likely to increase. A recent study shows that OSA affects 34% of men and 17% of women and is largely undiagnosed [8]. CPAP treatment, the gold standard therapy, exists but is often poorly tolerated for patients with OSA. Thus, the development of new or combinations of treatments are needed.

OSA is associated with increased cardiovascular morbidity and mortality, including hypertension, coronary heart disease, heart failure, pulmonary hypertension, and atrial fibrillation [1, 9]. OSA can cause intermittent hypoxia (IH), hypercapnia, and sleep fragmentation. IH is the main injury factor leading to cardiovascular morbidity and mortality [10, 11]. Sympathetic overactivation and systemic oxidative stress may be the main mechanisms associated with IH [1]. These abnormalities all contribute to the development of cardiovascular remodelling, including ventricular hypertrophy, endothelial dysfunction, carotid intima-media thickness, and alterations in the coronary microcirculation. The obvious modifications in cardiovascular remodelling are characterized by the disruption of normal myocardial structure through excessive collagen deposition. Our previous study confirms that OSA can induce cardiac perivascular fibrosis [12]. However, the detailed mechanisms remain unclear. Given the increased risk of cardiac remodelling in OSA patients, an improved understanding of the underlying mechanisms is necessary.

Different from terminally differentiated cells, vascular smooth muscle cells (VSMCs) own a distinctive ability of plasticity to alternate from a differentiated/contractile state, which expresses elevated levels of contractile proteins, such as *α*-smooth muscle actin (*α*-SMA), towards a dedifferentiated/synthetic state, which expresses increased levels of osteopontin (OPN) at different pathologic conditions [13, 14]. VSMC phenotypic switching is widely involved in atherosclerosis and research has proved that inhibiting VSMC phenotypic switching may be beneficial in atherosclerosis [14, 15]. Hypoxia increased the involved genes' expression in dedifferentiation and profibrosis of human bladder smooth muscle cells [16]. Hypoxia inducible factor-*α* (HIF-*α*) inhibition can decrease systemic vascular remodelling diseases [17]. However, no research reports show that VSMC phenotypic switching is involved in OSA-induced cardiac perivascular fibrosis, and its mechanisms remain to be further elucidated.

The PHD3, which is located in the cytoplasm [18], has been regarded as a cellular low oxygen sensor [19]. With sufficient oxygen availability, PHD3 can hydroxylate the HIF-*α* via von Hippel-Lindau protein (pVHL); however, it can maintain cell survival or proliferation functions under hypoxia [19]. PHD3 is different from other PHDs. First, PHD3 is abundantly expressed in the heart [20]. Second, PHD3 can retain its activity under prolonged mild hypoxia (2%–5%), so PHD3 has been deemed as the main regulator in prolonged and mild hypoxia [21]. Under hypoxia, PHD3 is found in high-order complexes. It is different from the aggregates which reveal under normoxia [22]. When PHD3 is released from the aggregates, it may be able to freely interact with its cytoplasmic and nuclear targets [23]. As a controversial protein, PHD3 maintains carcinoma cell growth [24], but other researchers also consider that the loss of PHD3 allows tumours to overcome hypoxic growth inhibition and sustain proliferation [25]. However, the function of PHD3 in VSMCs phenotypic switching has not been reported.

Based on these findings, we hypothesized that PHD3 may exhibit a protective effect on IH-induced cardiac microvascular fibrosis via inhibiting VSMCs phenotypic switching. To uncover the mechanisms, we explored its role in vitro and vivo.

## 2. Methods

The experiments conformed to the Guide for the Care and Use of Laboratory Animals published by the US National Institutes of Health. The study protocol was approved by the Institutional Ethics Committee of Shandong University and Southeast University.

### 2.1. Study Design

C57BL/6J wild-type (WT) male 6-week-old mice (21–23 g) were purchased from Yangzhou University (Yangzhou, China). A total of 60 mice were randomly assigned to normoxia or IH exposure for 1 week. After one week, the IH mice were randomly divided into 5 groups: IH, IH+shRNA PHD3 NC (shNC), IH+lentiviral PHD3 NC (LvNC), IH+shRNA PHD3, and IH+lentiviral PHD3 (we followed the method of Zhang et al. 2017).

### 2.2. IH

For 3 months, mice, which need to IH exposure, received 60 hypoxic events/h (20 s at 5% O_2_ followed by 40 s of room air) during 8 h/d, corresponding to severe OSA. Control mice with normoxic breathing were placed in an identical device, but the hypoxic gas was replaced by room air [12, 26, 27] (we followed the method of Zhang et al. 2017).

### 2.3. Histology and Immunohistochemistry

The preparation of section and immunohistochemistry analysis was performed as described [12]. For immunohistochemistry, sections were incubated with primary antibody against collagen I (1:1000 Abcam), collagen III (1:1000 Abcam) and *α*-SMA (1:200 Abcam), OPN (1:200 Abcam) overnight at 4°C and incubated with secondary antibody at 37°C for 30 min. The perivascular region fibrotic area was measured from all groups in every 6 randomly chosen views of each sample and analyzed by the Image-Pro Plus 6.0 program (we followed the method of Zhang et al. 2017).

### 2.4. Cell Culture

VSMCs were purchased from ScienCell Research Laboratories, Inc. (Carlsbad, CA). Dulbecco's modified Eagle's medium supplemented with 10% fetal bovine serum (FBS) and 2 mM glutamine was used. Cells were cultured in a humidified 5% CO_2_ and 95% air incubator at 37°C and 3-5 passages were used. Cells were treated with IH for 72 h. Air-phase set-point consisted of a 35-minute hypoxic period, followed by 25 minutes of reoxygenation (21% O_2_ and 5%CO_2_) [12, 28, 29] (we followed the method of Zhang et al. 2017). Before IH treatment, the VSMCs were infected with lentivirus at a multiplicity of infection of 15 for 24 h. Cells were treated with siRNA (GenePharma Shanghai, China) in order to inhibit HIF-1*α* expression. The target sequence for siHIF-1*α* was 5'-CCAUGUGACCAUGAGGAAATT-3', and the negative sequence was 5'-UUUCCUCAUGGUCACAUGGTT-3'.

### 2.5. Western Blot Analysis

Whole-cell proteins were isolated from freshly dissected mice hearts and cell lysates. Western blot analysis was performed as described [30]. Primary antibodies was used against PHD3 (1:500 Novus), HIF-1*α* (1:1000 Cell Signaling Technology), *α*-SMA (1:1000 Abcam), OPN (1:500 Abcam), collagen I (1:5000 Abcam), collagen III (1:5000 Abcam), and *β*-actin (1:2000 Abcam) (we followed the method of Zhang et al. 2017).

### 2.6. RNA Extraction and Quantitative Real-Time PCR

TRIZOL (TIANGEN) was used to extract total RNA from tissues or cultured VSMCs. Real-time PCR was performed using SYBR green reagent (TIANGEN) and ABI 7500 system. Target genes' primers were synthesized by TIANGEN Biotech (Beijing, China). Relative mean fold change in expression was calculated by the 2^-△△CT^ method.

### 2.7. Immunofluorescence Microscopy

In brief, after fixation and blocking, cells were incubated with primary antibody against *α*-SMA (1:200 Abcam), OPN (1:200 Abcam), and HIF-1*α* (1:200 Cell) at 4°C overnight and incubated with secondary antibody for 30 min at 37°C (Alexa Fluor 488 and 549 goat anti-rabbit; Alexa Fluor 549 and 488 goat anti-mouse 1:400). Nuclei were counterstained with 4′-6-diamidino-2-phenylindole (DAPI). Specific fluorescence was acquired by fluorescence microscope (IX-71, Olympus) (we followed the method of Zhang et al. 2017).

### 2.8. Statistical Analysis

Results are from 3 repeated experiments. Data are reported as the mean ± SD. Results were compared by 2-tailed Student t-test for 2 groups and one-way ANOVA followed by Tukey's t-test (2-tailed) for multiple groups. SPSS (v16.0; SPSS, Inc, Chicago, IL) was used for analysis. Differences were considered statistically significant at* p* < 0.05.

## 3. Result

### 3.1. IH Induced Collagen Deposition

**Figure 1:**
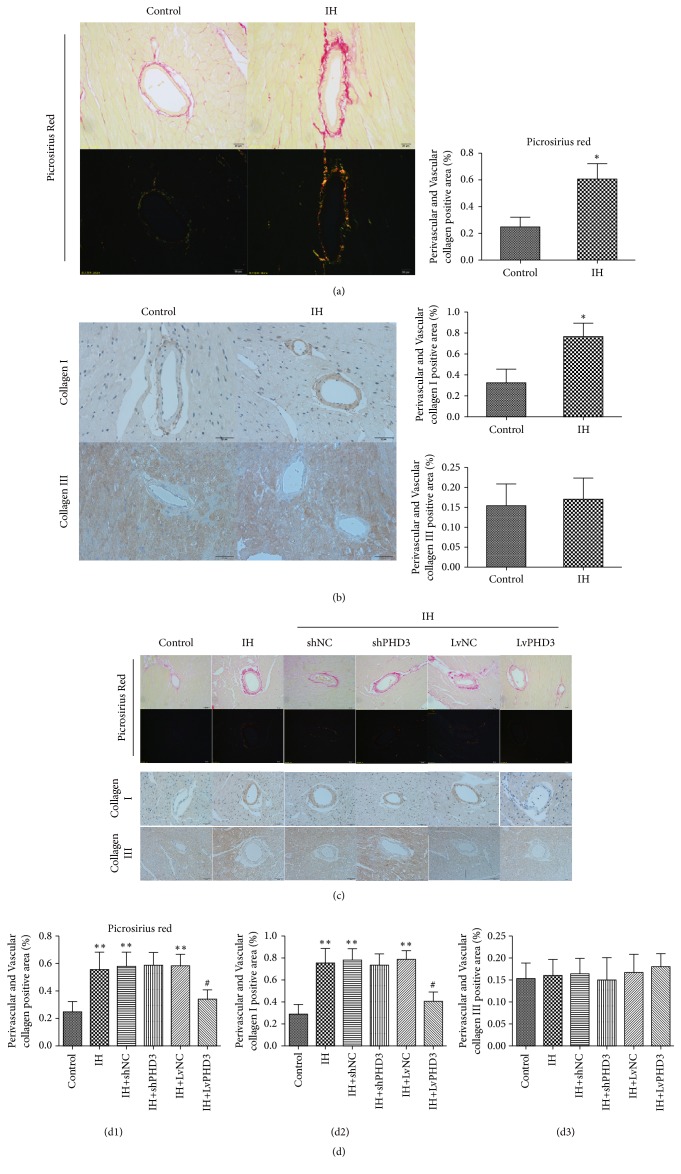
*IH induces cardiac perivascular fibrosis and PHD3 overexpression improves it*. (a) Representative images of the heart section with Picrosirius Red staining (red, yellow, and green staining) for each group (original magnification ×400 bars=20 *μ*m). And quantification of Picrosirius red staining. (b) Representative images of the heart section with immunohistochemical staining of collagen I and collagen III (yellow staining) for each group (original magnification ×400 bars=20 *μ*m). Quantification of collagen I and collagen III. Data are mean ± SD; n=10 per group. *∗p*<0.05 versus control. (c) Representative images of the heart section with Picrosirius red staining (red, yellow, and green staining) and immunohistochemical staining of collagen I and collagen III (yellow staining) for each group (original magnification ×400 bars=20 *μ*m). (d) Quantification of Picrosirius red staining (d1), collagen I (d2), and collagen III (d3). Data are mean ± SD; n=10 per group. *∗∗p*<0.01 versus control; #*p*<0.05 versus IH+LvNC.

To ascertain whether IH can induce cardiac fibrosis, histochemistry stain was used. Picrosirius red staining revealed greater collagen deposit in the region of perivascular and vascular of the IH than control. Quantitative analysis showed a 2.4-fold increase in IH group as compared with the control group (0.57 ± 0.11 versus 0.24 ± 0.07,* p*<0.05) (Figure 1(a)). Immunohistochemistry showed that IH enhanced collagen I expression when compared with the control group (0.76 ± 0.12 versus 0.32 ± 0.13,* p*<0.05) (Figure 1(b)). However, IH exposure had no effect on the expression of collagen III (*p*>0.05) (Figure 1(b)). Interestingly, compared with control, IH exposure did not obviously increase collagen deposition in myocardium (Supplementary Figure 1). In other words, the region of IH-induced collagen deposition only existed in cardiac perivascular and vascular tissues.

### 3.2. PHD3 Overexpression Improves IH-Induced Cardiac Perivascular Fibrosis

Our previous study had shown that IH can induce PHD3 expression in vitro and vivo [12]. To ascertain whether PHD3 could improve cardiac fibrosis induced by IH, in this study, lentivirus was applied to intervene the expression of PHD3 in vivo and vitro. Transfection efficiency was evaluated by GFP, which reached values of up to 70% in vivo and 90% in vitro (Supplementary Figure 2B). Western blot revealed that lentiviral vector can increase or decrease PHD3 expression obviously compared with NC group in vivo and vitro (Supplementary Figures 2A and 2B).

Picrosirius red staining indicated greater collagen deposit in the area of perivascular and vascular of the IH group than control. And quantitative analysis showed a 2.29-fold increase in IH group as compared with control mice (0.55 ± 0.13 versus 0.25 ± 0.07,* p*<0.01) (Figures 1(c) and 1(d1)). LvPHD3 treatment improved collagen deposition as compared with LvNC group (0.34 ± 0.06 versus 0.58 ± 0.07,* p*<0.05) (Figures 1(c) and 1(d1)). However, shPHD3 treatment could not improve collagen deposition as compared with shNC group (*p*>0.05) (Figures 1(c) and 1(d1)). For further verification, immunohistochemical staining of collagen was used. Immunohistochemical staining indicated that IH exposure increased collagen I expression as compared to the control (0.75 ± 0.13 versus 0.28 ± 0.08,* p*<0.01) (Figures 1(c) and 1(d2)). However, LvPHD3 significantly improved the increased expression as compared with LvNC (0.4±0.08 versus 0.78±0.08,* p*<0.05) (Figures 1(c) and 1(d2)). Our study showed that shPHD3 treatment did not change collagen I expression as compared with shNC (*p*>0.05) (Figures 1(c) and 1(d2)). Interestingly, we discovered that IH did not seem to affect collagen III expression (*p*>0.05) (Figures 1(c) and 1(d3)).

### 3.3. IH Induces VSMC Phenotypic Switching

VSMC phenotypic switching has been shown to contribute to vascular remodelling. To explore whether IH induced VSMC phenotypic switching, we performed in vitro and in vivo experiments.

**Figure 2:**
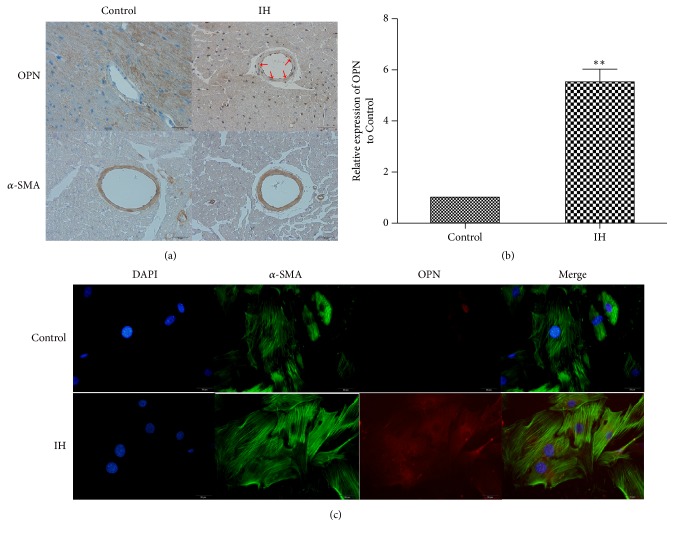
*IH induces VSMC phenotypic switching in vivo and vitro*. (a) Representative images of immunohistochemical staining of OPN and *α*-SMA (yellow staining) in the perivascular and vascular region (original magnification ×400 bars=20 *μ*m). (b) Quantification of immunohistochemical staining of OPN in vivo. (c) Representative images of double immunofluorescence staining with antibodies to *α*-SMA (green) and OPN (Red). Nuclei were counterstained with DAPI (blue) (original magnification ×400 bars=20 *μ*m). Data are mean ± SD; n=10 per group. *∗∗p*<0.01 versus control.

In vivo, *α*-SMA, a specificity marker of differentiated/contractile state of VSMCs, was assayed by immunohistochemistry. The area of *α*-SMA expression was restricted to the vascular media in both control and IH group. OPN, a specificity marker of dedifferentiated/synthetic state of VSMCs, was not expressed in vascular media in the control group. However, after IH treatment, the expression of OPN dramatically increased in vascular media compared with the control (5.5 ± 0.5 versus 1,* p*<0.01) (Figures 2(a) and 2(b)) (red arrow). Furthermore, we used immunofluorescence-colocalized staining to detect VSMC phenotypic switching in vitro. We demonstrated that IH exposure significantly increased the expression of OPN, which is consistent with the study in vivo (Figure 2(c)).

### 3.4. PHD3 Overexpression Improves IH-Induced VSMC Phenotypic Switching

**Figure 3:**
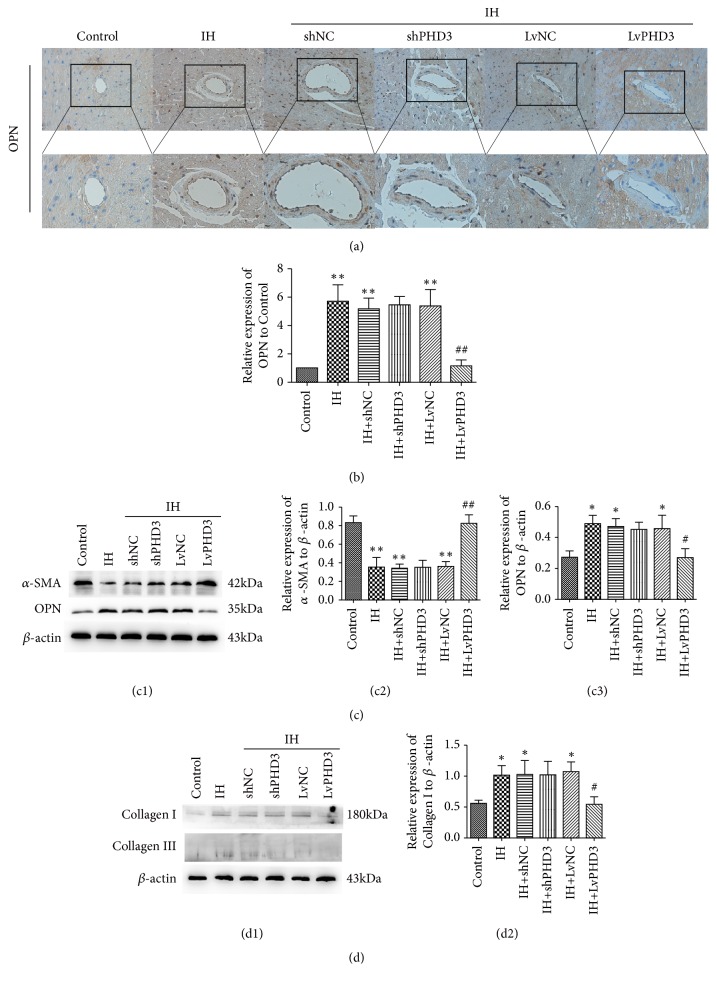
*PHD3 overexpression improved IH-induced VSMCs phenotypic switching*. (a) Representative images of immunohistochemical staining of OPN (yellow staining) in the perivascular and vascular region (original magnification ×400 bars=20 *μ*m). (b) Quantification of immunohistochemical staining of OPN in vivo. (c) Western blot analysis and quantification of *α*-SMA (c1 c2) and OPN (c1 c3) in vitro. (d) Western blot analysis and quantification of collagen I (d1 d2) and collagen III (d1). Data are mean ± SD; *∗p*<0.05 versus control; *∗∗p*<0.01 versus control; #*p*<0.05 versus IH+LvNC; ##*p*<0.01 versus IH+LvNC.

To further explore the mechanism which PHD3 overexpression improves IH induced cardiovascular fibrosis, we designed the next experiments. In vivo, OPN was detected by immunohistochemistry. It revealed that OPN expression dramatically increased in vascular media after IH treatment compared with control (5.7 ± 1.1 versus 1,* p*<0.01) (Figures 3(a) and 3(b)). However, LvPHD3 improved OPN expression as compared with LvNC group (1.13 ± 0.4 versus 5.36 ± 1.2,* p*<0.01) (Figures 3(a) and 3(b)). However, shPHD3 cannot decrease OPN expression compared with vehicle treatment (*p*>0.05) (Figures 3(a) and 3(b)). In vitro, Western blot analysis demonstrated that IH exposure significantly increased the expression of dedifferentiated/synthetic state marker OPN (0.49 ± 0.05 versus 0.27 ± 0.04,* p*<0.05) (Figures 3(c1) and 3(c3)) and decreased the expression of differentiated/contractile state markers *α*-SMA (0.35 ± 0.09 versus 0.83 ± 0.07,* p*<0.01) (Figures 3(c1) and 3(c2)) as compared with the control. However, the phenotypic switching above were improved when PHD3 was overexpressed: OPN (0.26 ± 0.05 versus 0.45 ± 0.08,* p*<0.05) (Figures 3(c1) and 3(c3)) and *α*-SMA (0.82 ± 0.09 versus 0.36 ± 0.05,* p*<0.01) (Figures 3(c1) and 3(c2)). ShPHD3 cannot improve the features above (*p*>0.05) (Figure 3(c)). When VSMCs experienced phenotypic switching, we found that collagen I expression increased compared with control (1.02 ± 0.16 versus 0.56 ± 0.05,* p*<0.05) (Figure 3(d)). However, compared with LvNC group, collagen I expression decreased in LvPHD3 group (0.54 ± 0.11 versus 1.08 ± 0.15,* p*<0.05) (Figure 3(d)). Similarly, compared with shNC group, collagen I expression was not improved in shPHD3 group (*p*>0.05) (Figure 3(d)). The WB indicated that VSMC phenotypic switching may not influence the expression of collagen III (*p*>0.05) (Figure 3(d)).

### 3.5. PHD3 Overexpression Accelerates HIF-1*α* Degradation in VSMCs

**Figure 4:**
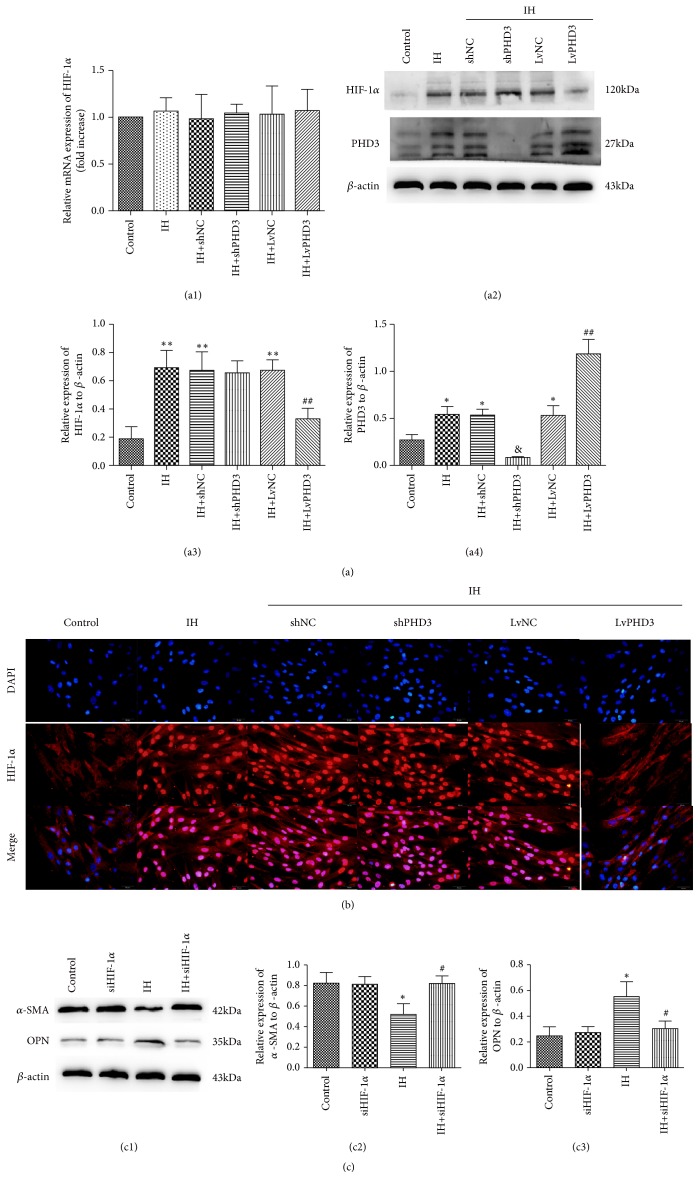
*PHD3 overexpression degrades and inactivates HIF-1α*. (a) RT-qPCR analysis of HIF-1*α* mRNA (a1), Western blot analysis and quantification of HIF-1*α* (a2 a3), and PHD3 (a2 a4). (b) Representative images of immunofluorescence staining with antibodies to HIF-1*α* (red). Nuclei were counterstained with DAPI (blue) (original magnification ×400 bars=20 *μ*m). (c) Western blot analysis and quantitative analysis of *α*-SMA (c1 c2), OPN (c1 c3). *∗p*<0.05 versus control; *∗∗p*<0.01 versus control; ##*p*<0.01 versus IH+LvNC; #*p*<0.05 versus IH;* p*<0.05 versus IH+shNC.

To further clarify the mechanism of PHD3 function in VSMCs phenotypic switching, we explored the potential signal transduction pathways involved in IH-induced cardiac fibrosis. In vitro, WB revealed that PHD3 expression increased after IH treatment compared to control (0.54 ± 0.08 versus 0.27 ± 0.06,* p*<0.05) (Figures 4(a2) and 4(a4)). Under IH, lentiviral vector could increase or decrease PHD3 expression compared with NC group (0.08 ± 0.01 versus 0.53 ± 0.06* p*<0.05, 1.18 ± 0.15 versus 0.53±0.1* p*<0.01) (Figures 4(a2) and 4(a4)). RT-qPCR indicated that HIF-1*α* mRNA cannot be affected by IH, shPHD3, and LVPHD3 (*p*>0.05) (Figure 4(a1)). We found that compared with control, the expression of HIF-1*α* increased after IH exposure (0.69 ± 0.12 versus 0.19 ± 0.08* p*<0.01) (Figures 4(a2) and 4(a3)), but LVPHD3 can improve it as compared with LVNC (0.33 ± 0.07 versus 0.67 ± 0.07* p*<0.01) (Figures 4(a2) and 4(a3)). Compared with shNC, no change in shPHD3 group was found (*p*>0.05) (Figures 4(a2) and 4(a3)). Furthermore, immunofluorescence indicated that IH resulted in HIF-1*α*'s relocation (Figure 4(b)) from cytoplasm to nucleus, which means that HIF-1*α* is activated by IH. Compared with LvNC, LvPHD3 can maintain most HIF-1*α* (Figure 4(b)) in the cytoplasm, but shPHD3 was incapable of inactivating HIF-1*α* (Figure 4(b)) as compared with shNC. To further clarify, we used siRNA to inhibit HIF-1*α*. Western blot revealed that siRNA can decrease HIF-1*α* expression compared with NC group in vitro (Supplementary Figure 2C). Compared with IH, siHIF-1*α* can improve IH-induced VSMCs phenotypic switching: *α*-SMA (0.82 ± 0.06 versus 0.52 ± 0.1* p*<0.05) and OPN (0.3 ± 0.06 versus 0.55 ± 0.11* p*<0.05) (Figure 4(c)). From the above results, IH activates HIF-1*α* and induces VSMC phenotypic switching. PHD3 can improve the switching via accelerating HIF-1*α* degradation.

## 4. Discussion

In the present study, we consider that PHD3 improves the early cardiovascular remodelling induced by OSA. In the field of experimental OSA, considerable studies have focused on the effects of IH, but few studies have explored a novel therapeutic target for preventing early cardiovascular remodelling in patients with OSA. To our knowledge, this research is the first study showing that VSMC phenotypic switching is the basis for the progression of OSA-induced cardiovascular remodelling. Our research identifies a previously undiscovered function of PHD3 in OSA-induced cardiovascular remodelling. Our novel experiment revealed that PHD3 owns a protective role in IH-induced cardiovascular fibrosis by inhibiting smooth muscle dedifferentiation.

OSA induces various pathophysiological triggers, but IH plays the most pivotal role in the development of cardiovascular diseases [31]. Some research has demonstrated beneficial effects in animal models with IH short-term exposure [32]. However, long-term exposure (at least 4 weeks) often causes detrimental effects [33]. In the present study, we evaluated the level of cardiovascular fibrosis resulting from IH for 3 months, which can mimic severe OSA in patients. After IH exposure (3 months), immunohistochemical staining indicates that IH induced perivascular and vascular media fibrosis; however, it had no effect on myocardial interstitial fibrosis, which is in accordance with the previous study [34]. Interestingly, compared with the control, the expression of collagen I was significantly increased after IH exposure, but there is no significant difference in collagen III expression in vivo. Perivascular fibrosis, which can reduce the elasticity of microvessels, increases the vascular wall thickness and plays an important role in the development of heart failure [35]. In the present study, we did not access the cardiac function because our previous study indicated that cardiac function decreased after IH exposure [12]. PHD3, a cellular oxygen sensor, is not an activator of cell apoptosis but is a promoter of cell survival under restricted oxygen [19]. PHD3 is thought to be the most important regulator of HIF-1*α* under severe and prolonged hypoxia compared with other PHDs. Under hypoxia, besides HIF, PHD3 may possess other hydroxylation targets, such as ATF4, PK-M2, and Pax2 [19]. In our previous study, PHD3 expression was upregulated after IH treatment in vivo and vitro, but its function may be limited. After interference and overexpression, we found that the fibrosis improved when PHD3 was overexpressed. Thus, we concluded that PHD3 improves IH-induced perivascular and vascular media fibrosis. To elucidate its mechanism, we designed the experiment in vivo and vitro.

Vascular remodelling is dependent on dynamic interactions between many pathological factors. IH is deemed as an independent factor which promotes vascular remodeling [36]. As we know, VSMCs phenotypic switching plays a key role in the processes of multiple vascular pathologies [37]. Different from the terminally differentiated cells, VSMCs own a distinctive ability of plasticity that allows the phenotypic shift from a differentiated/contractile state to a dedifferentiated/synthetic state [14]. In contractile state, VSMCs are characterized by low proliferation rates and low rates of protein synthesis [38]. However, in the synthetic state, VSMCs are characterized by relatively low contractile protein expression, re-entry into the cell cycle, high level of proliferation and migration, and high rates of protein synthesis and secretion [38]. VSMCs could easily switch between these two states when remodelling is required. A disruption of the balance, for example, the synthetic phenotype predominates, may be an underlying cause of many vascular diseases. With respect to VSMC phenotypic switching, most of the researchers paid their attention on atherosclerosis and aneurysms, and little is known regarding it on OSA-induced cardiovascular remodelling. Therefore, we first evaluated the switching of VSMCs phenotype during OSA-induced cardiovascular remodelling and found that the expression of OPN, a marker of synthetic state, was remarkably upregulated in microvascular media in vivo. To further demonstrate our observations, we designed experiments in vitro. VSMCs increased the expression of OPN after IH treatment. Interestingly, a subtle paradox was present here: the expression of *α*-SMA showed no change in vivo and decreased in vitro. The disparity in results may reflect differences in vivo, which was affected by multiple factors, and in vitro, which was only affected by the single factor. In vitro, we also found that collagen I expression and not collagen III increased after IH-induced VSMCs phenotypic switching. This finding can explain why IH only induced collagen I to deposit in perivascular and vascular media in vivo.

We found another important finding in our study. PHD3 improved IH-induced cardiac microvascular fibrosis via inhibiting VSMC dedifferentiation in vivo and vitro. In our study, we indicated that VSMC dedifferentiation was involved in IH-induced cardiac microvascular fibrosis. Next, we used lentivirus to change PHD3 expression. We found that PHD3 overexpression can improve IH-induced VSMCs dedifferentiation in vivo and vitro. However, shPHD3 cannot improve it. In our study, we demonstrated that PHD3 may be a novel marker which has the capacity to modulate VSMCs phenotypic switching for the first time.

We further explored the molecular mechanism of PHD3 in vitro. Shan, F. indicated that HIF-1*α* was involved in the phenotypic modulation in pulmonary artery SMCs during hypoxia [39]. Lambert, C. M. indicated that HIF-1 inhibition could decrease systemic vascular remodelling diseases [17]. In our present study, qPCR revealed no change of HIF-1*α* mRNA in IH/shPHD3/LVPHD3 compared with the control. WB revealed that HIF-1*α* expression increased after IH exposure compared to control, but LvPHD3 can improve the increased expression. Immunofluorescence indicated that IH resulted in HIF-1*α*'s relocation from cytoplasm to nucleus, and LvPHD3 can maintain most HIF-1*α* in the cytoplasm. To further clarify, we used siRNA to inhibit HIF-1*α*. We found that siHIF-1*α* can improve IH-induced VSMC phenotypic switching. Thus, we concluded that PHD3 improves VSMC dedifferentiation via degrading and inactivating HIF-1*α* and by not reducing generation. The axis of PHD3-HIF-1*α*-OPN/*α*-SMA may be involved in the improvement of VSMC dedifferentiation. Another study indicated that OPN enabled transcriptional upregulation of HIF-1a expression under both normoxia and hypoxia [40]. A plausible explanation for the controversy between these results might lie in the versatility and diversity of HIF-1*α* and OPN functions and the intrinsic divergence in the research subjects.

Several limitations are present in our research. (1) OSA results in IH, hypercapnia. All the negative factors could lead to cardiovascular remodelling. We only focused on IH. It is a limitation because the IH model does not represent OSA. However, IH is the most powerful factor in OSA-induced negative factors. Complications, such as cardiac remodelling and heart failure, were reported in our previous research [12]. Furthermore, the model has been used to explore OSA and its complications, such as cardiac remodelling, by international and domestic academicians [27]. (2) Cultured VSMCs can switch phenotype when they were passaged, which can cause potential bias in vitro. However, in each experiment, we have tried our best to use VSMCs from the same passage which might reduce the bias. (3) We used human aortic VSMCs to explore the mechanism in vitro. Mouse coronary micro-VSMCs in vitro are a reasonable option. We finally selected human cells because mouse coronary micro-VSMCs are difficult to extract and cannot be purchased from companies. Furthermore, PHD3 is an ortholog in both humans and mice. Hence, we firmly believe that human aortic VSMCs can replace mouse coronary micro-VSMCs in vitro study. (4) We used lentivirus to change the expression of PHD3, and its effect is not better than knockout or transgenic technology. However, in our study, the lentivirus basically achieved the anticipated effect.

## 5. Conclusions

We first found that PHD3 can improve cardiac perivascular fibrosis by inhibiting VSMC phenotypic switching in an OSA mice model. The mechanism may be involved in degrading and inactivating HIF-1*α*. Although the exact underlying mechanisms are not fully understood, given the cardioprotective effects of PHD3 overexpression, PHD3 may be a potential therapeutic target for OSA-induced heart diseases.

## Data Availability

The data used to support the findings of this study are included within the article.

## Abbreviations

CIH: Chronic intermittent hypoxia
CPAP: Continuous positive airway pressure
HIF-1*α*: Hypoxia inducible factor-1*α*
IH: Intermittent hypoxia
OPN: Osteopontin
OSA: Obstructive sleep apnea
PHD3: Prolyl 4-hydroxylase domain protein 3
SMAs: Smooth muscle cells
VSMCs: Vascular smooth muscle cells
WT: Wild-type.

## Supplementary Materials

Supplementary Materials Supplementary Figure 1: Picrosirius Red and collagen I and collagen III staining in myocardium region. Representative images of the heart section with Picrosirius Red staining (red, yellow, and green staining), and immunohistochemical staining of collagen I and collagen III (yellow staining) for each group (original magnification ×400 bars=20*μ*m). Supplementary Figure 2: change of PHD3/HIF-1*α* expression by lentivirus/siRNA transfection. (A) Representative image of GFP-labeled lentivirus in cardiac tissues by fluorescence microscopy (original magnification ×100 bar=50*μ*m) (a1). Western blot analysis and quantitative analysis of PHD3 (a2 a3) in vivo. (B) Representative image of GFP-labeled lentivirus in VSMCs by fluorescence microscopy (original magnification ×100 bar=50*μ*m) (b1). Western blot analysis and quantitative analysis of PHD3 (b2 b3) in vitro. (C) Western blot analysis and quantitative analysis of HIF-1*α* (c1 c2) in vitro after siRNA treatment. Data are mean±SD; n=10 per group. *∗*p<0.05 versus shNC or ##p<0.01 versus LvNC.
