# Supplementary material for: Prolyl 4-Hydroxylase Domain Protein 3-Inhibited Smooth-Muscle-Cell Dedifferentiation Improves Cardiac Perivascular Fibrosis Induced by Obstructive Sleep Apnea
Source: Biomed Res Int. 2019 Jun 27;2019:9174218. doi: 10.1155/2019/9174218 (PMC6621170; doi:10.1155/2019/9174218)

**Legends for Supplementary Figures:**

**Supplementary Figure 1. Picrosirius Red and collagen I III staining in myocardium region**

Representative images of the heart section with Picrosirius Red staining (red, yellow and green staining), and immunohistochemical staining of collagen I collagen III (yellow staining) for each group. (original magnification ×400 bars=20μm).

**Supplementary Figure 2. Change of PHD3/HIF-1α expression by lentivirus/siRNA transfection**

**(A)** Representative image of GFP-labeled lentivirus in cardiac tissues by fluorescence microscopy (original magnification ×100 bar=50μm) (a1). Western blot analysis and quantitative of PHD3 (a2 a3) in vivo. **(B)** Representative image of GFP-labeled lentivirus in VSMCs by fluorescence microscopy (original magnification ×100 bar=50μm) (b1). Western blot analysis and quantitative of PHD3 (b2 b3) in vitro. **(C)** Western blot analysis and quantitative of HIF-1α (c1 c2) in vitro after siRNA treatment. Data are mean±SD; n=10 per group. **p*<0.05 vs shNC or ##*p*<0.01 vs LvNC.

**Supplementary Figure 1**


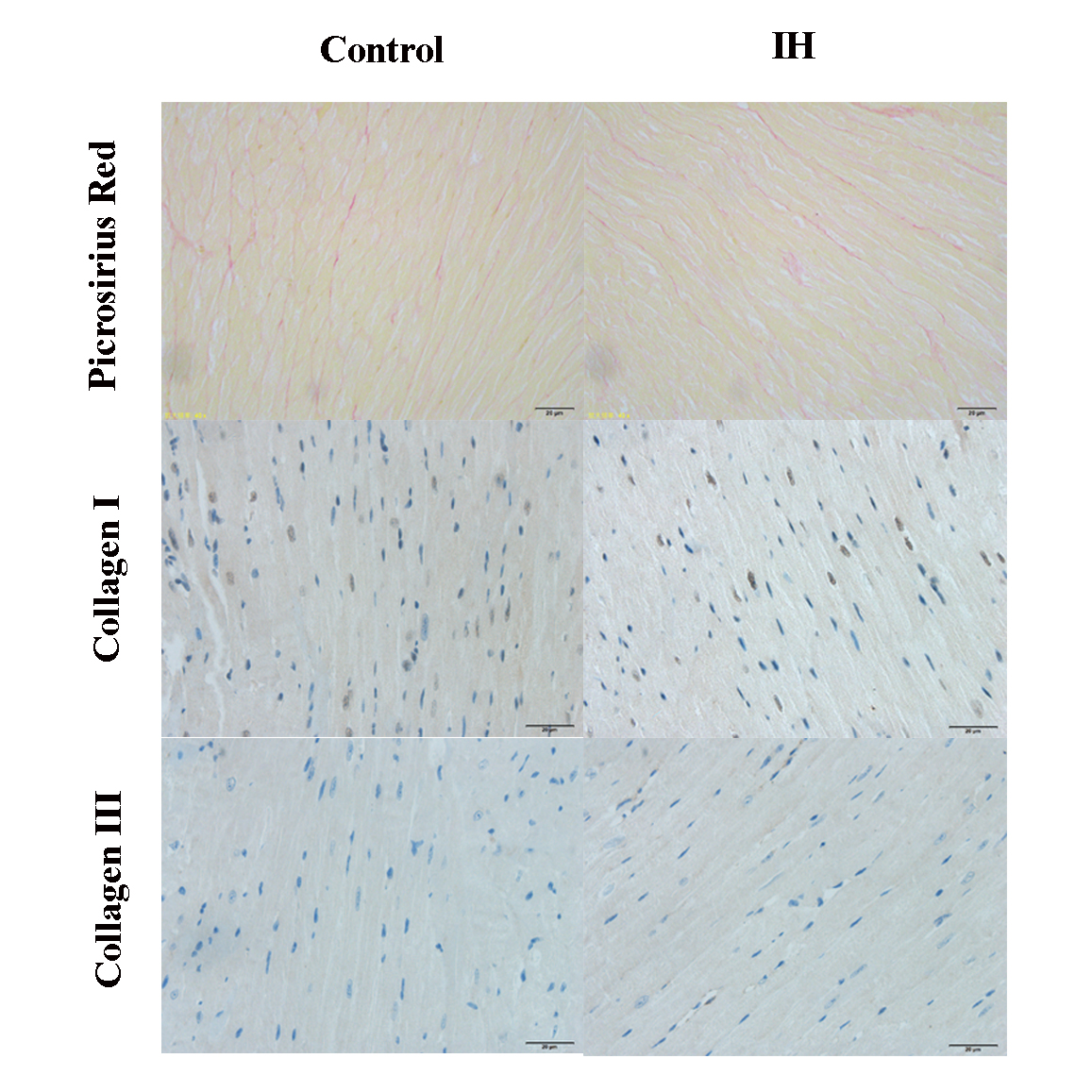


**Supplementary Figure 2**


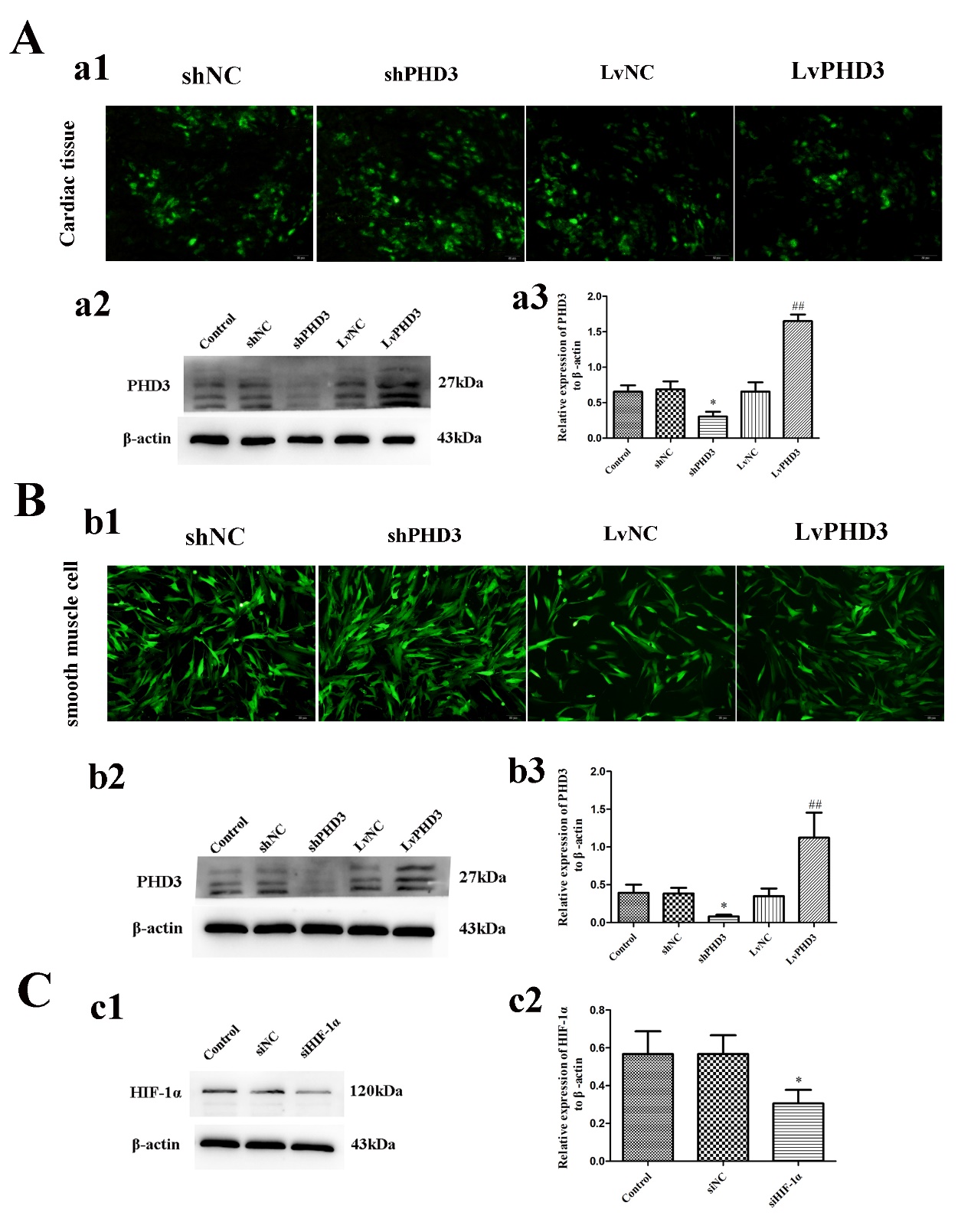

Supplement: Supplementary Materials — Supplementary Figure 1: Picrosirius Red and collagen I and collagen III staining in myocardium region. Representative images of the heart section with Picrosirius Red staining (red, yellow, and green staining), and immunohistochemical staining of collagen I and collagen III (yellow staining) for each group (original magnification ×400 bars=20μm). Supplementary Figure 2: change of PHD3/HIF-1α expression by lentivirus/siRNA transfection. (A) Representative image of GFP-labeled lentivirus in cardiac tissues by fluorescence microscopy (original magnification ×100 bar=50μm) (a1). Western blot analysis and quantitative analysis of PHD3 (a2 a3) in vivo. (B) Representative image of GFP-labeled lentivirus in VSMCs by fluorescence microscopy (original magnification ×100 bar=50μm) (b1). Western blot analysis and quantitative analysis of PHD3 (b2 b3) in vitro. (C) Western blot analysis and quantitative analysis of HIF-1α (c1 c2) in vitro after siRNA treatment. Data are mean±SD; n=10 per group. ∗p<0.05 versus shNC or ##p<0.01 versus LvNC. [file 9174218.f1.docx]
